# Minimally invasive percutaneous new designed transpedicular lag-screw fixation for the management of Hangman fracture using O-arm-based navigation: a clinical study

**DOI:** 10.1186/s12891-023-06614-4

**Published:** 2023-06-15

**Authors:** Yijie Liu, Xuefeng Li, Tangyiheng Chen, Jie Chen, Yi Zhu, Genglei Chu, Huilin Yang, Weimin Jiang

**Affiliations:** 1 Department of Orthopaedic Surgery, The First Affiliated Hospital of Soochow University, 899 Pinghai Street, Suzhou, 215006 China; 2 Department of Orthopaedic Surgery, Dushu Lake Hospital Affiliated to Soochow University, 9 Chongwen Road, Suzhou, 215124 Jiangsu Province China

**Keywords:** Hangman fracture, Minimally invasive, New transpedicular lag-screw, O-arm navigation

## Abstract

**Background:**

To investigate the outcomes and safety of using minimally invasive percutaneous new transpedicular lag-screw fixation with intraoperative, full rotation, three-dimensional image (O-arm)-based navigation for the management of Hangman fracture.

**Methods:**

Twenty-two patients with Hangman fracture were treated with minimally invasive percutaneous new transpedicular lag-screws using intraoperative, full rotation, and three-dimensional image (O-arm)-based navigation. The preoperative and postoperative conditions of the patients were evaluated according to the ASIA (American Spinal Injury Association) scale. The patient's VAS (visual analog scale) scores before and after surgery, operation time, cervical vertebral activity, intervertebral angle and bone healing were recorded and collected, and repeated measures analysis of variance was used for statistical analysis.

**Results:**

All patients were satisfactorily repositioned after surgery, and the VAS scores for neck pain were significantly lower than those before surgery on the first day and at 1 month, 3 months and the last follow-up (*P* < 0.001). According to the ASIA scale, four patients recovered from preoperative grade D to postoperative grade E. Bony fusion was achieved for all cases, and the range of neck rotation was restored to normal at the last follow-up. The post-surgery angular displacement (AD) demonstrated the stability of C2-3 after our new screw fixation for the treatment of Hangman fracture.

**Conclusions:**

Minimally invasive percutaneous new transpedicular lag-screw fixation using intraoperative, full rotation, three-dimensional image (O-arm)-based navigation achieved satisfactory clinical results with the advantages of immediate stability, safety and effectivity. We suggest that it is a reliable and advanced technique for the management of Hangman fracture.

## Introduction

Hangman fracture, also known as traumatic spondylolisthesis of C2, refers to a fracture occurring between the superior and inferior articular processes under trauma mostly caused by falling and traffic accidents. It was first described by Wood Jones in 1913 and then defined and named by Schneider in 1965 based on the similarities of the fractures seen after judicial hanging [1].

Hangman fracture is divided into four types based on the most widely accepted Levine-Edward classification [2]. While most type I Hangman fractures can be treated conservatively, there is still controversy regarding the treatment of unstable types II, IIA and III Hangman fractures [3]. With the advancement of clinical application anatomy and the maturity of upper cervical spine surgery techniques, many scholars advocate early surgical treatment [4]. The C2 transpedicular screw fixation proposed by Judet in 1964 is called physiological reconstruction because of its low trauma, immediate stability and maximum retention of the physiological functions of adjacent segments [5]. However, the common, nondedicated pedicle lag-screw has the disadvantages of insufficient reduction, poor fixation stability and proneness to overpressurization. Based on the above problems, we have developed a new type of transpedicular lag-screw. The transpedicular lag-screw has been biomechanically tested, and the results show that its performance is superior to that of ordinary lag-screws [6]. On the premise of good clinical outcomes using this new type of C2 pedicle lag-screw for treatment of Hangman fracture with open surgery [7], 22 cases of Hangman fracture were treated with minimally invasive percutaneous new transpedicular lag-screw fixation in the present study. The aim of this study was to investigate the outcomes and safety of using minimally invasive percutaneous new designed transpedicular lag-screw fixation with intraoperative, full rotation, three-dimensional image (O-arm)-based navigation for the management of Hangman fracture.

## Materials and methods

This study is a retrospective, single-center, open-label case series. The study was approved by Institutional Ethics Committee of Soochow University (ethical code 2020179), and all methods were performed in accordance with the Declaration of Helsinki. Written informed consent was obtained from all individual participants. The inclusion criteria included the following: (1) Hangman fracture with satisfactory reduction after traction treatment, fracture end separation ≤ 2 mm, and no obvious angulation; (2) preoperative cervical magnetic resonance imaging (MRI) indicating no spinal cord compression at the C2-3 level; (3) no severe osteoporosis. The exclusion criteria included the following: (1) pedicle of C2 developmental malformation; (2) bilateral pedicle comminuted fracture; and (3) infection in the operative field and other factors affecting the operation.

From October 2015 to February 2021, 22 patients including 15 males and 7 females with Hangman fracture were treated with minimally invasive percutaneous new transpedicular lag-screw fixation using intraoperative, full rotation, three-dimensional image (O-arm)-based navigation. The age of the patients ranged from 24 to 83 years, with an average of 48.6 ± 15.7 years. There were 15 cases (68.2%) caused by car accidents and 7 cases (31.8%) by fall injuries. According to the Levine-Edwards classification [2], 18 cases were type II and 4 cases were type IIA. All 22 patients had neck pain and limited neck motion. According to the American Spinal Injury Association (ASIA) score, 18 patients were in grade E, and 4 patients were in grade D. Imaging examination showed that 13 cases (59%) were simple Hangman fracture, 3 cases (14%) were complicated with type I atlas fracture, 2 cases (9%) with type II odontoid fracture, 1 case (4.5%) with type III odontoid fracture, 1 case (4.5%) with spleen rupture, and 2 case (9%) with rib fracture and hemopneumothorax. Three of the 22 patients (14%) had an atypical Hangman fracture with one side of the fracture line at the junction of the pedicle and the vertebral body with the other side at the lamina. The remaining 19 patients’ (86%) fracture lines were located in the bilateral pedicles. No cervical spinal cord compression was found in all cases from preoperative MRI. The patients’ pre and postoperative data are shown in Table 1.

**Table 1:** Data of 22 patients

Case	Age/Sex	Injury	Levine and Edwards classification	Preoperative ASIA grade	Operative time (min)	Postoperative ASIA grade	Follow-up duration(months)
1	34 yr/F	MVA	TypeII	E	50	E	12
2	43 yr/M	Fall	TypeII	E	63	E	12
3	51 yr/F	MVA	TypeIIA	E	72	E	24
4	53 yr/M	Fall	TypeII	E	60	E	24
5	83 yr/M	MVA	TypeII	D	75	E	36
6	71 yr/F	MVA	TypeII	E	50	E	36
7	54 yr/F	Fall	TypeII	E	85	E	24
8	41 yr/M	MVA	TypeII	E	72	E	24
9	24 yr/M	Fall	TypeII	D	55	E	36
10	35 yr/M	MVA	TypeIIA	E	66	E	36
11	52 yr/F	MVA	TypeII	E	62	E	36
12	40 yr/M	MVA	TypeII	E	90	E	24
13	60 yr/M	MVA	TypeII	D	57	E	24
14	28 yr/F	MVA	TypeII	E	50	E	36
15	47 yr/F	Fall	TypeIIA	E	85	E	24
16	40 yr/M	MVA	TypeII	E	55	E	36
17	39 yr/M	Fall	TypeIIA	D	75	E	36
18	65 yr/M	MVA	TypeII	E	58	E	24
19	25 yr/M	Fall	TypeII	E	70	E	24
20	62 yr/M	MVA	TypeII	E	62	E	24
21	49 yr/M	MVA	TypeII	E	80	E	12
22	72 yr/M	MVA	TypeII	E	63	E	36

*ASIA* American Spinal Injury Association, *MVA* motor vehicle accident, *M* male, *F* female

### New transpedicular lag-screw

**Fig. 1:**
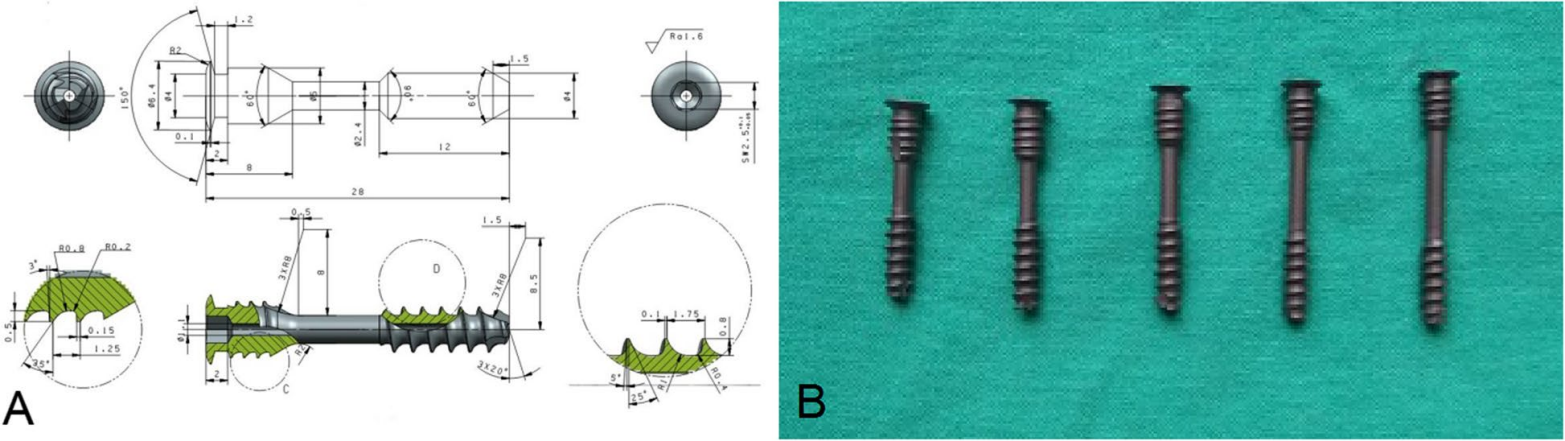
Picture **A** is a schematic image of the new transpedicular lag-screw. Picture **B** is an image of the new transpedicular lag-screw

This screw is a double-threaded hollow lag-screw specifically designed for Hangman fractures based on a large number of anatomical and imaging measurements. The length of the new transpedicular lag-screw ranges from 22–32 mm, the diameter of the head thread is 4 mm, the tail thread measures 5 mm, and the screw fastener measures 6.4 mm. The pitch of the head thread and tail thread is 1.75 mm and 1.25 mm, respectively. The rotation of the head thread over the fracture line theoretically produces a 0.5 mm reduction pressurization of the fracture end, and the screw tail cap prevents the screw from sinking into the bone surface. Due to the length of the screw and the limitation of the tail cap, excessive pressurization can be avoided, and it can compress the fracture twice (Fig. 1).

### Operative procedure and perioperative managements

All patients underwent skull traction with weight from 2 to 3 kg and a traction time of 5 to 7 days. Lateral cervical radiograph was regularly checked to adjust the traction weight and angle according to the traction effect and reset condition. All cases achieved a satisfactory position, that is, the fracture end separation was no more than 2 mm, and there was no obvious angle. Preoperative cervical radiographs were combined with CT three-dimensional reconstruction for anatomical measurement of the bony structure. MRI was performed at the same time to understand the integrity of the adjacent disc and the anterior and posterior longitudinal ligaments.

**Fig. 2:**
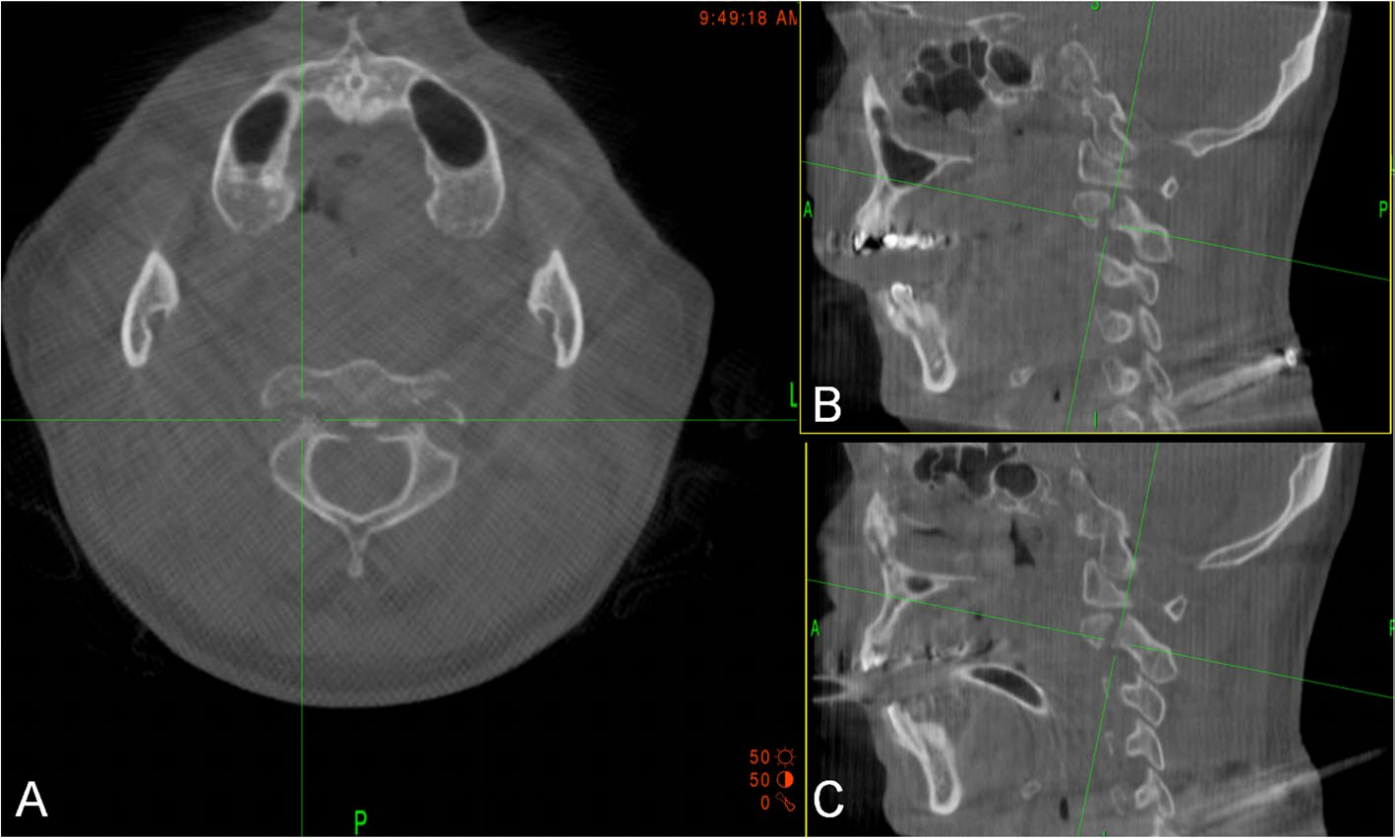
Intraoperative CT scan was automatically registered to the image guidance system, which showing fracture of pars on both the sides on parasagittal sections (**A**, **B**, **C**)

**Fig. 3:**
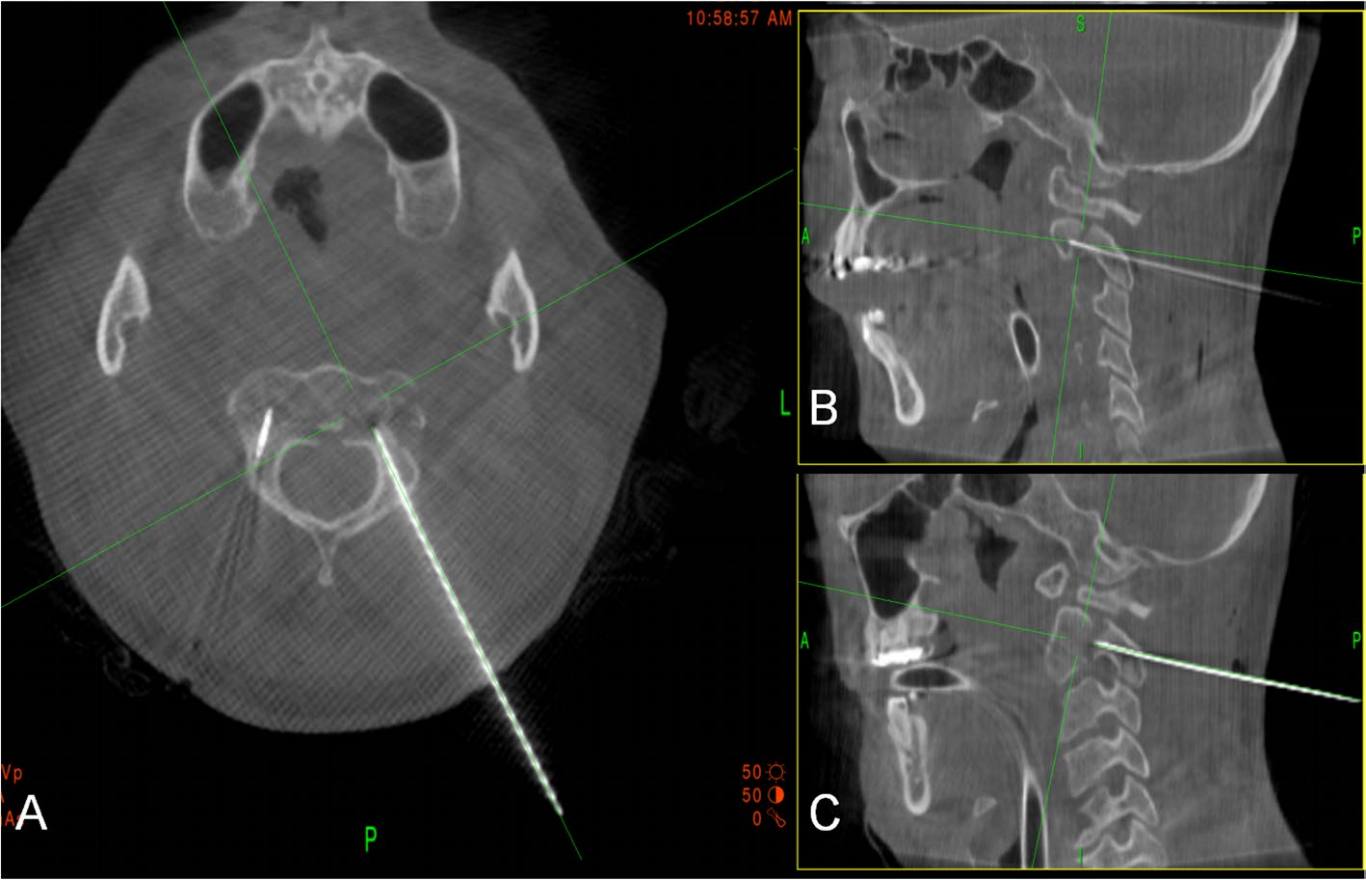
Intraoperative computed tomographic (CT) scans showing right-sided K-wire in axial and sagittal planes in C2 pedicle (**A**, **B**). Intraoperative CT scans showing left-sided K-wire in axial and sagittal planes in C2 pedicles (**A**, **C**)

**Fig. 4:**
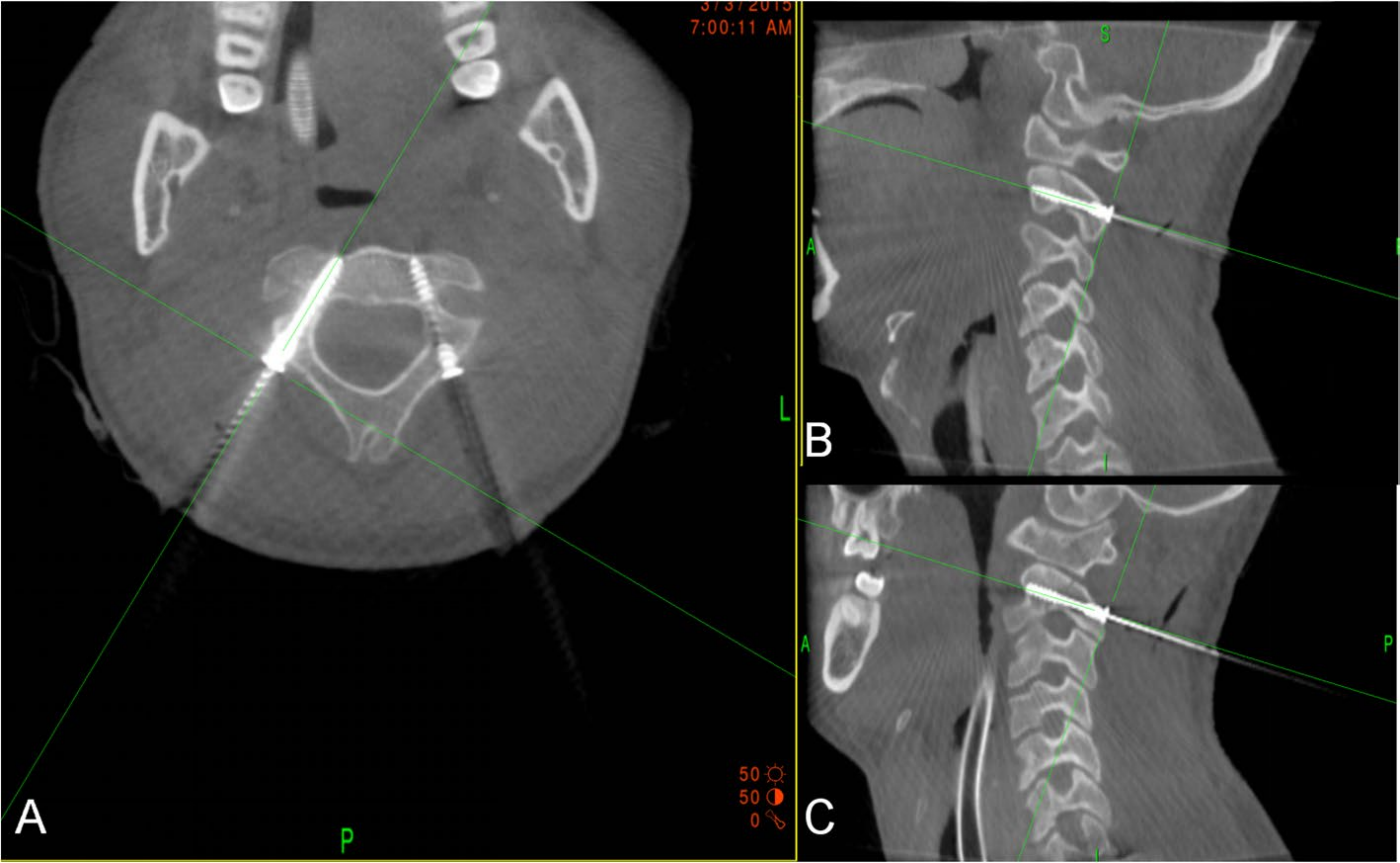
Intraoperative computed tomographic CT scans showing right-sided C2 pedicle screw in axial and sagittal planes (**A**, **C**). Intraoperative CT scans showing left-sided C2 pedicle screw in axial and sagittal planes (**A**, **B**)

Surgeries were all performed by one experienced surgeon. The patient underwent general anesthesia and then was placed in a prone position on a Jackson radiolucent table with skull traction and then sterilely prepped and draped. After installing a neurophysiological monitoring device, the O-arm navigation system was used to obtain 3D reconstructed images to confirm the fracture reduction. The reference frame of 3-Dimentional navigation system was placed on the spinous process of C5 through an isolated small incision, and the intraoperative CT scan was automatically registered to the image guidance system (Fig. 2). The navigation probe determined the skin entry point, approximately 2 cm on bilateral sides of the spinous process, and made a 1 cm incision. Minimally invasive tools were used to establish the working channel. After using the infrared navigation probe to confirm the position and angle of the entry point, a 1.0 mm diameter K-wire was inserted with a drill under navigation (Fig. 3). After reconfirming the position and angle, the surgeon started tapping and then inserting a pedicle lag-screw with the appropriate length to gradually pressurize and reset the position, which was noted in order to stop the skull traction before pressurizing and resetting (Fig. 4). Routine closure was carried out after the position of the screws was finally confirmed by O-arm scan and reconstruction.

Antibiotics were not routinely used after the operation. Patients with neurological deficit before surgery were given intravenous neurotrophic drugs. If the patients had no other combined injuries, they were allowed to get up with a neck rigid collar on the first day after surgery. Additionally, they were reassessed through cervical lateral films, open-mouth films and three-dimensional reconstructions of CT scan images. Rigid collar was instructed to be worn for 2 weeks.

### Data collection and outcome evaluation

Routine follow-up was performed at postoperative one month, three months and six months and then every six months thereafter. Lateral and open-mouth radiographs of cervical vertebrae were reexamined one month after the operation, dynamic radiographs of cervical vertebrae and three-dimensional reconstructions of CT scan images were reexamined at three months; patients who did not achieve osseous union in 3 months follow-up were asked to follow up once a month thereafter. Neck pain was assessed by a VAS score from 0 (painless) to 10 (severe pain), and neurological function was graded by the ASIA scale before and after the operation. Fracture healing was evaluated according to radiograph and CT sagittal reconstruction. The standard of healing was as follows: the fracture line disappeared, the local bone trabecula and bone cortex were continuous, and there was no obvious instability on dynamic positioned radiograph. The accuracy of screw placement was classified using a modified classification of Gertzbein and Robbins^8^: grade 1 (screw completely within the pedicle), grade 2 (< 50% of the screw diameter outside the pedicle), and grade 3 (> 50% of the screw diameter outside the pedicle). The results of the follow-ups were recorded and evaluated by the two physicians in our team.

The neck range of motion (ROM) is measured by the overall movement of the neck in different directions: flexion, extension, lateral flexion, and rotation to both sides. The patient should be seated or standing comfortably with their back straight and shoulders relaxed. Flexion: Have the patient slowly move their chin towards their chest, keeping their eyes forward. Measure the angle between their chin and their chest. Extension: Have the patient move their head back as far as possible while keeping their chin level. Measure the angle between their head and their chin. Lateral flexion: Have the patient slowly move their ear towards their shoulder. Measure the angle they achieve with each side. Rotation: Have the patient slowly rotate their head as far as possible to each side. Measure the angle they achieve with each side.

The intervertebral disc angle of C2-3 is formed by the lines parallel to the superior endplate of C2 and the inferior endplate of C3 in overflexional and overextensive X-rays, with the neck positioned in maximum flexion and extension, respectively. Angular displacement (AD) is the change in angle between C2-3 in dynamic lateral overflexional and overextensive views.

### Statistics

Data entry and statistical analysis were performed using the Statistical Package for the Social Sciences software (SPSS 20.0, USA). The measured data are expressed as mean ± SD. Comparisons of clinical and radiological outcomes pre and postoperatively were performed using repeated measurement analysis of variance. Differences were considered statistically significant when *p* < 0.05.

## Results

The surgeries were performed successfully in all cases. The operative time ranged from 50 to 90 min and the average was 66.1 ± 11.9 min. There were 19 cases with bilateral fixation and 3 cases with single side fixation. The indication for unilateral stabilization is atypical Hangman fracture with one side of the fracture line at the junction of the pedicle and the vertebral body with the other side at the lamina. All 22 patients were followed up for 12 to 36 months with an average of 27.3 ± 8.4 months. The neck pain of all patients significantly decreased. The preoperative VAS score was 6.7 ± 1.2 points and decreased to 2.6 ± 1.1 points on the first day, to 1.3 ± 0.5 points after 1 month, to 1.1 ± 0.4 points after 3 months and to 1.1 ± 0.6 points at the last follow-up. The VAS scores at the first day, 1 month, 3 months, and the last follow-up were statistically significant compared to those before the operation (*P* < 0.001, Table 2), while the difference between the last follow-up and 3 months follow-up was not statistically significant (*P* = 0.75, Table 2). According to the ASIA scale, 4 patients recovered from preoperative grade D to grade E.

**Table 2:** The mean VAS scores before operation and during follow-up (mean ± SD)

Parameters	VAS Scores	*P* value
Preoperative	6.7 ± 1.2	
Postoperative 1 day	2.6 ± 1.1^a^	< 0.001
Postoperative 1 month	1.3 ± 0.5^a^	< 0.001
Postoperative 3 months	1.1 ± 0.4^a^	< 0.001
Final follow-up	1.1 ± 0.6^b^	0.75

*VAS* Visual Analogue Scale; ^a^*P* < .05 comparing with preoperative value; ^b^*P* > .05 comparing with postoperative 3 months

The 3-month postoperative cervical vertebrae activity was still partially restricted, considering that the fracture had not healed. The 6-month postoperative cervical vertebrae activity was significantly different from that at 3 months postoperatively, indicating that the ROM was obvious improved (*P* < 0.001). At the last follow-up, the ROM was not significantly different from that at 6 months after surgery, indicating that all patients had no loss of ROM. The ROM data are summarized in Table 3.

**Table 3:** The neck range of motion (ROM) during follow-ups (degrees, mean ± SD)

Parameters	Flexion	Extension	Left flexion	Right flexion	Left rotation	Right rotation
Postoperative 3 months	24.7 ± 3.0	26.1 ± 2.7	24.9 ± 4.1	25.4 ± 3.6	52.7 ± 6.6	52.0 ± 6.1
Postoperative 6 months	37.8 ± 2.1^a^	39.5 ± 1.7^a^	39.5 ± 2.1^a^	40.2 ± 1.8^a^	71.1 ± 2.7^a^	70.2 ± 2.4^a^
Final Follow-up	38.2 ± 1.9^b^	40.1 ± 1.6^b^	40.3 ± 1.4^b^	41.0 ± 1.6^b^	71.2 ± 2.2^b^	70.8 ± 1.6^b^
^a^*p*	< 0.001	< 0.001	< 0.001	< 0.001	< 0.001	< 0.001
^b^*p*	0.17	0.22	0.07	0.13	0.58	0.33

^a^*P* value comparing with postoperative 3 months; ^b^*P* comparing with postoperative 6 months

Cervical stability refers to the ability of cervical spine to maintain its normal alignment and resist excessive movements that may lead to injury or damage. The intervertebral angle (C2-3) of flexion–extension position and angular displacement (AD) at 3 months, 6 months, and the final follow-up was recorded and analyzed for any abnormalities or changes in C2-3. AD is the change in angle between C2-3 in dynamic lateral overflexional and overextensive views. Our results exhibited no obvious AD (< 11°) of C2-3 during the follow-up time, and no significant differences in intervertebral angle and AD were noted during the follow-ups. The post-surgery AD demonstrated the stability of C2-3 after our new screw fixation for the treatment of Hangman fracture, which may decrease the risk of further injury or damage (Table 4).

**Table 4:** The intervertebral angle (C2-3) during follow-ups (degrees, mean ± SD)

Parameters	Over flexion position	Over extension position	AD
Postoperative 3 months	4.5 ± 1.7	-0.6 ± 0.8	5.1 ± 2.1
Postoperative 6 months	4.4 ± 1.6^a^	-0.7 ± 0.6^a^	5.1 ± 1.7^a^
Final Follow-up	4.5 ± 1.7^b^	-0.5 ± 0.5^b^	5.0 ± 2.0^b^
^a^*P*	0.67	0.72	0.85
^*b*^*P*	0.80	0.10	0.69

*AD* Angular displacement; ^a^*P* value comparing with postoperative 3 months; ^b^*P* comparing with postoperative 6 months

Osseous union was achieved in 14 patients at the 3-month follow-up. Five patients (3 patients with atlas fractures and 2 patients with odontoid fractures) achieved osseous union at the 5-month follow-up. Two patients (1 patient with spleen rupture, 1 patient with multiple rib fractures and blood pneumothorax) achieved osseous union at the 6-month follow-up. One patient with odontoid fracture (posterior pedicle screw combined with anterior odontoid screw fixation) achieved osseous union at the 9-month follow-up. The average union time among 22 patients was 4.0 ± 1.6 months.

**Fig. 5:**
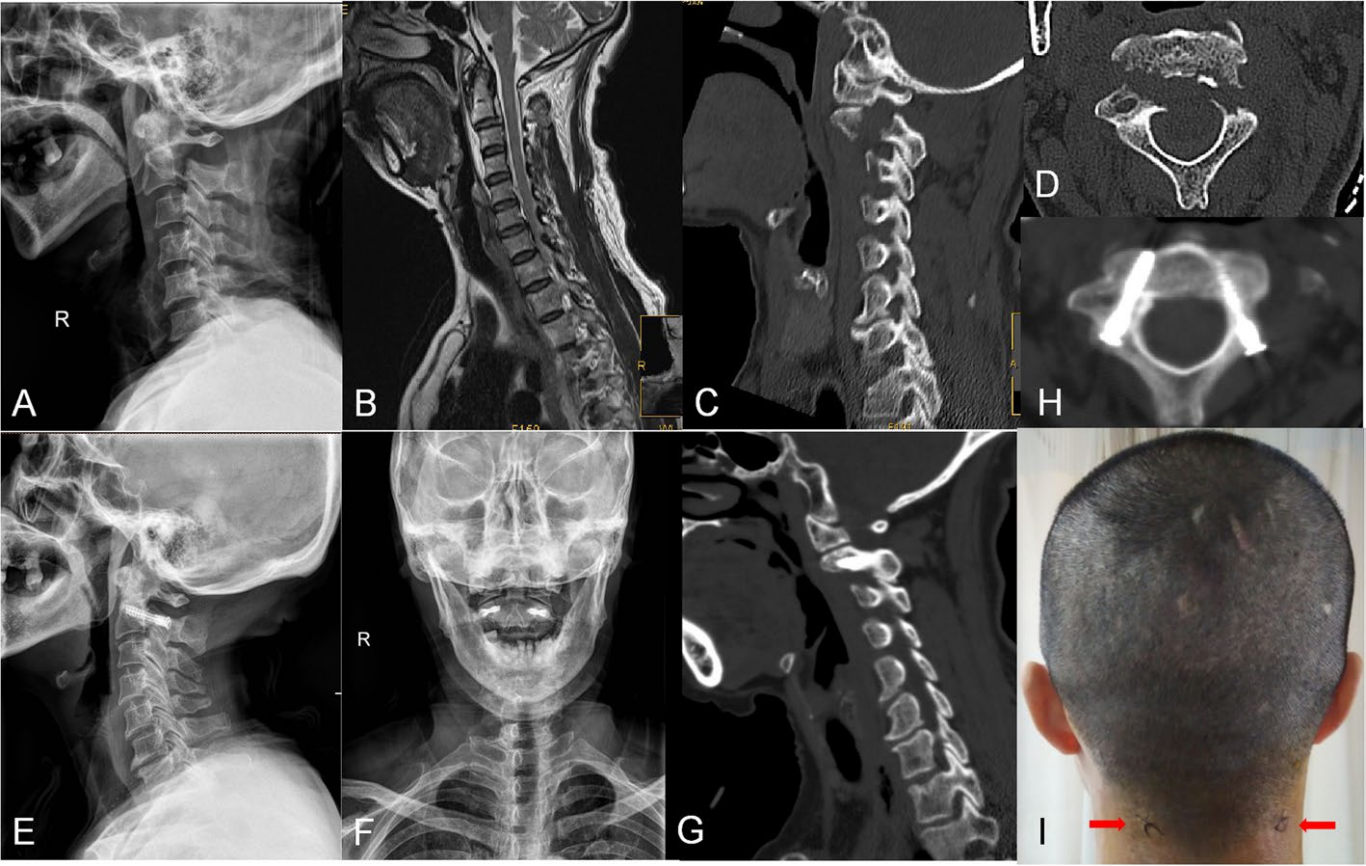
The pre-operative lateral radiographs of a 35-year-old male patient with a type II Hangman fracture (**A**). Pre-operative sagittal T2-weighted magnetic resonance image revealed the absence of spinal cord compression (**B**). Pre-operative computed tomography (CT) scan reconstructions show obvious separation of fracture on both the sides(**C**, **D**). The post-operative open-mouth and lateral radiographs showed adequate fracture reduction (**E**, **F**). The computed tomography (CT) scan reconstructions at the 3-month follow-up showed excellent fusion (**G**, **H**). The minimal incisions of this technology (**I**)

None of the 22 patients sustained spinal cord or vertebral artery injuries during the operation, and there were no complications such as postoperative infection, internal fixation failure, cervical deformity, or fracture relocation. 41 screws were grade 1, 1 screw was grade 2 because of the corresponding C2 pedicle developmental narrowness according to the modified classification of Gertzbein and Robbins. 1 patient developed intermittent neck pain 1 month after surgery, which was considered to be related to excessive cervical motion. This patient's neck pain decreased after wearing a rigid collar for another two weeks, and osseous union was also achieved by the 3-month follow-up (Fig. 5).

## Discussion

In 1964, Judet used C2 transpedicular screw fixation for the first time to treat Hangman fracture [5]. It was found that this treatment can immediately restore and pressurize the fracture and preserve the maximum rotation function between C1 and C2 and the physiological function between C2 and C3. This technique was called “physiological reconstruction” and was widely used thereafter.

Type I Hangman fractures are generally considered stable fractures and can be treated with a hard neck brace or Halo-vest [8, 9]. However, with the continuous accumulation and follow-up of medical records, complications such as nonunion, pseudoarticular formation and kyphosis caused by conservative treatment are receiving more attention [10]. In addition, the relatively bulky external fixation braces and long-term skull traction have brought many inconveniences to the patients' lives. Therefore, many scholars also advocate early minimally invasive surgical treatment for even type I Hangman fractures [11]. For type II, type IIA and type III unstable Hangman fractures, surgery is currently considered the preferred treatment option. There is still controversy over the choice of surgical approach [3–7, 12]. Although anterior surgery can yield good results, which has been reported by many scholars [13], the high anterior approach may endanger important structures, especially the facial and hypoglossal nerves, branches of the external carotid artery, and contents of the carotid sheath [14]. Moreover, this procedure cannot solve dislocation of the posterior arch of C2, which may pose approach-related problems. The posterior approach was associated with a relatively simple exposure with no involvement of major vascular and visceral structures as well as a lower complication rate. However, both anterior cervical discectomy and fusion (ACDF) and C2-3 screw fixation will cause mobility loss in the fusion segment [15]. Posterior C2 transpedicular screw fixation is advocated by many scholars, and the results of open surgery confirm that it is an effective surgical procedure [9, 16].

This fixation of C2 can preserve motion of normal segments, which is considered to be a “physiologic operation”. However, the side effects of a regular C2 transpedicular screw, such as incomplete reduction, screw dislodgement, and excessive compression, are still unavoidable when direct pars repair is performed for patients. To reduce the potential complications, a new C2 transpedicular lag-screw was designed by our team. This new transpedicular lag-screw is a double-thread screw based on a Herbert screw. The diameter of the head thread is less than the tail thread, and the corresponding pitch of the head thread is longer than the pitch of the tail thread. This unique structure not only can offer a double-fixation mechanism that is superior to common lag-screws with single fixation, but also can avoid excessive pressurization. The new transpedicular lag-screw has been used in human cadaver spines for biomechanical testing, and the results showed that the biomechanical properties of the new C2 transpedicular lag-screw were better than ordinary screws [6]. Accordingly, we used this new type of C2 pedicle lag-screw for treatment of Hangman fracture with open surgery, which achieved satisfactory results [7].

As we know the traditional open C2 transpedicular screw fixation technique still has many disadvantages, such as extensive exposure, early postoperative pain, and neck muscle denervation and atrophy, which makes the minimally invasive concept more important in high-segment cervical surgery. In surgical management, posterior direct repair of C2 using the trans-pedicular screw through the minimal invasive approach has recently been described [17–19]. However, one of the most significant challenges of minimally invasive spine surgery is the lack of standard anatomic landmarks encountered during conventional open spine surgery, which reduces the information available to the surgeon; hence, the surgeon can become disoriented and ‘‘lost’’ in the spine. The intraoperative, full rotation, three-dimensional image (O-arm)-based navigation system can obtain high-quality 3D CT images in a short time and be directly input into the navigation computer for automatic matching and registration so that the surgeon can accurately complete the operation under the "direct view". Yoshida et al. [20] reported a case of percutaneous C2 pedicle lag-screw use in the treatment of Hangman fracture with the O-arm navigation system and proposed that the minimally invasive percutaneous technique is worthy of promotion. To the best of our knowledge, there is still no clinical study of minimally invasive percutaneous transpedicular lag-screw fixation using intraoperative, full rotation, three-dimensional image (O-arm)-based navigation for the management of Hangman fracture, especially, we used the new transpedicular screw in all cases. The minimally invasive procedure presented here seems to be equally safe and effective as the open procedures previously reported. In addition, it has many other advantages: (1) it is a kind of minimally invasive surgery, which can also achieve a satisfactory fusion rate by using a new transpedicular lag-screw; (2) the safety is greatly improved under navigation and neurological monitoring; (3) it can achieve early postoperative recovery, which avoids complications of open surgery and long-term bed rest. In our study, all patients received the above benefits of minimally invasive surgery.

Compared with traditional conservative treatments, such as traction and bracing, minimally invasive surgery for Hangman fracture has obvious advantages in safety, reliability and immediate stability. We believe that this procedure can also be performed on type I Hangman fracture with the consent of the patient. This procedure is theoretically applicable to all cases of replaceable Hangman fracture. As the new transpedicular lag-screw has limited reduction ability, good reduction must be obtained by skull traction before surgery. This technique is not applicable to Hangman fractures in which there are vertebral pedicle developmental deformities, fracture lines that are too oblique and bilateral pedicle comminuted fractures. Because of the poor self-repairing ability of intervertebral discs, Hangman fractures combined with intervertebral disc injury will cause of C2-3 intervertebral instability. For those patients with Hangman fractures combined with slight disc injuries and no obvious herniated disc, this new technique was still recommended for the treatment of Levine-Edward type II fractures as a safe and effective technique. The post-surgery AD (< 11°) demonstrated the stability of C2-3 after our new screw fixation for the treatment of Hangman fracture, which may decrease the risk of further injury or damage. However, examination of patients by preoperative cervical MRI demonstrated that an intervertebral disc with obvious rupture and spinal compression should be carefully considered before treatment with open surgery such as anterior cervical discectomy and fusion or posterior C2-3 fixation and fusion surgery.

This study has several limitations. First of all, the number of cases in this study is relatively small and some of the cases’ follow-up time are short. Experience with a greater number of patients and long-term follow-up is still necessary to further evaluate this technique. Another limitation is that this study is an uncontrolled case series which only emphasized the feasibility, safety and effectiveness of the minimally invasive surgery. A further prospective study comparing minimally invasive surgery with open surgery and conservative treatment would be carried out.

## Conclusion

In the treatment of patients with Hangman fractures, minimally invasive percutaneous new transpedicular lag-screw fixation using intraoperative, full rotation, three-dimensional image (O-arm)-based navigation is a minimally invasive, safe and effective technique.

## Data Availability

Written informed consent was obtained from all individual participants for publication of artitle and any accompanying images. A copy of the written consent is available for review by the Editor-in-Chief of this journal. The datasets used and/or analyzed during the current study are available from the corresponding author on reasonable request.

## References

[1] Schneider RC, Livingston KE, Cave AJ (1965). “Hangman’s fracture” of the cervical spine. J Neuro surg.

[2] Levine AM, Edwards CC (1985). The management of traumatic spondylolisthesis of the axis. J Bone Joint Surg Am.

[3] Prost S, Barrey C, Blondel B (2019). Hangman’s fracture: Management strategy and healing rate in a prospective multi-centre observational study of 34 patients. Orthop Traumatol Surg Res.

[4] Choi Man Kyu, Kwak Youngseok, Kim Ki Hong, et al. Direct trans-pedicular screw fixation for atypical hangman's fracture: a minimally invasive technique using the tubular retractor system. J Clin Neurosci*.* 2019;70:146–150. 10.1016/j.jocn.2019.08.046 31431401

[5] Judet R, Roy-Camille R (1970). Saillant G Actuallities de chiirugie orthopedique de 1’hopital Raymound-pointcare: fracture du rachis cervical.

[6] Huang L, Jiang WM, Luo ZP (2013). The design and biomechanical testing of a new transpedicular lag-screw for treatment of Hangman’s fracture (In Chinese, English abstract). Chin J Orthop.

[7] Liu Y, Zhu Y, Li X, Chen J, Yang S, Yang H, Jiang W (2020). A new transpedicular lag screw fixation for treatment of unstable Hangman's fracture: a minimum 2-year follow-up study. J Orthop Surg Res.

[8] Longo UG, Denaro L, Campi S (2010). Upper cervical spine injuries: indications and limits of the conservative management in Halo vest. A systematic review of efficacy and safety. Injury.

[9] Pravin Salunke, Sushanta K Sahoo, Prasad Krishnan, et al. Are C2 pars-pedicle screws alone for type II Hangman’s fracture overrated? Clin Neurol Neurosurg*.* 2016;141:7–12. 10.1016/j.clineuro.2015.11.019 26716722

[10] Rajasekaran S, Tubaki VR, Shetty AP (2012). Results of direct repair of type 2 hangman fracture using Iso-C3D navigation: 20 cases. J Spinal Disord Tech.

[11] Sugimoto Y, Ito Y, Shimokawa T (2010). Percutaneous screw fifixation for traumatic spondylolisthesis of the axis using iso-C3D fluoroscopy-assisted navigation (case report). Minim Invasive Neurosurg.

[12] Pankaj Kumar Singh, Kanwaljeet Garg, Duttaraj Sawarkar, et al. Computed tomography-guided C2 pedicle screw placement for treatment of unstable hangman fractures. Spine (Phila Pa 1976)*.* 2014;39:1058–65. 10.1097/BRS.0000000000000451 25122548

[13] Hao Xu, Zhao J, Yuan J (2010). Anterior discectomy and fusion with internal fixation for unstable hangman’s fracture. Int Orthop.

[14] Hur H, Lee JK, Jang JW (2014). Is it feasible to treat unstable hangman’s fracture via the primary standard anterior retropharyngeal approach?. Eur Spine J.

[15] Wang S, Wang Q, Yang H (2017). A novel technique for unstable Hangman's fracture: lag screw-rod (LSR) technique. Eur Spine J.

[16] ElMiligui Y, Koptan W, Emran I (2010). Transpedicular screw fixation for type II Hangman’s fracture: a motion preserving procedure. Eur Spine J.

[17] Lang Zhao, Tian Wei, Liu Yajun (2016). Minimally invasive pedicle screw fixation using intraoperative 3-dimensional fluoroscopy-based navigation (CAMISS Technique) for hangman fracture. Spine (Phila Pa 1976)..

[18] Kantelhardt SR, Keric N, Conrad J, Archavlis E, Giese A (2016). Minimally invasive instrumentation of uncomplicated cervical fractures. Eur Spine J.

[19] Avery Lee Buchholz, Steven L Morgan, Leslie C Robinson, et al. Minimally invasive percutaneous screw fixation of traumatic spondylolisthesis of the axis. J Neurosurg Spine*.* 2015;22:459–65. 10.3171/2014.10.SPINE131168 25723118

[20] Yoshida G, Kanemura T, Ishikawa Y (2012). Percutaneous pedicle screw fixation of a hangman's fracture using intraoperative, full rotation, three-dimensional image (O-arm)-based navigation: a technical case report. Asian Spine J.

